
JOURNAL INFORMATION
==============================
NLM Title Abbreviation: Biospektrum (Heidelb)
Journal ID: BIOspektrum (Heidelb.)
NLM Journal ID: 9615685

Biospektrum

ISSN: 0947-0867
EISSN: 1868-6249

ARTICLE INFORMATION
==============================
PMCID: PMC7474495
PMID: 32921927
DOI: 10.1007/s12268-020-1440-0
Article ID: 1440
Article version: 1
Subjects: Biotechnologie

Patientenantikörper als Informationsquelle

Falk Fabian
Zhou Zekun
Klinkusch Andreas
Weber Laura
Breitling Frank Frank.Breitling@KIT.edu

grid.7892.4 0000 0001 0075 5874 AG Peptidarrays, Institut für Mikrostrukturtechnik (IMT), Karlsruher Institut für Technologie, Herrmann-von-Helmholtz-Platz 1, D-76344 Eggenstein-Leopoldshafen, Deutschland
Electronic publication date: 2020 Sep 5
Print publication date: 2020
Volume: 26
Issue: 5
First page: 556
Last page: 558
Copyright: © Der/die Autor(en) 2020
License: Open Access Dieser Artikel wird unter der Creative Commons Namensnennung 4.0 International Lizenz veröffentlicht, welche die Nutzung, Vervielfältigung, Bearbeitung, Verbreitung und Wiedergabe in jeglichem Medium und Format erlaubt, sofern Sie den/die ursprünglichen Autor(en) und die Quelle ordnungsgemäß nennen, einen Link zur Creative Commons Lizenz beifügen und angeben, ob Änderungen vorgenommen wurden.
Die in diesem Artikel enthaltenen Bilder und sonstiges Drittmaterial unterliegen ebenfalls der genannten Creative Commons Lizenz, sofern sich aus der Abbildungslegende nichts anderes ergibt. Sofern das betreffende Material nicht unter der genannten Creative Commons Lizenz steht und die betreffende Handlung nicht nach gesetzlichen Vorschriften erlaubt ist, ist für die oben aufgeführten Weiterverwendungen des Materials die Einwilligung des jeweiligen Rechteinhabers einzuholen.
Weitere Details zur Lizenz entnehmen Sie bitte der Lizenzinformation auf http://creativecommons.org/licenses/by/4.0/deed.de.
License URL: https://creativecommons.org/licenses/by/4.0/

issue-copyright-statement: © Springer-Verlag GmbH Deutschland, ein Teil von Springer Nature 2020

==============================
Since decades antibodies are used for diagnosis e. g. by detecting patient antibodies that specifically bind to Influenza virus proteins. We predict these diagnostic questions will be parallelized to diagnose all known disease specific antibodies at once. These tests will ask in addition, which unknown antibodies patrol in a patient’s blood, and what exactly they bind to. Thereby, we expect to find antibody species that correlate to hitherto enigmatic diseases or have specialized functions.

■ Die Corona-Pandemie zeigt gerade, wie wichtig das Wissen ist, welche Antikörper ein Mensch entwickelt. Ein zur Diagnose eingesetztes Peptid oder Protein sollte möglichst alle infizierten Patienten entdecken und nur selten einen Fehlalarm auslösen. Für einen Impfstoff oder auch für eine passive Immunisierung mit Serumantikörpern sollten wir wissen, welche Antikörper den Patienten schützen bzw. welche ihm schaden (**Abb. 1**). Einige Stämme des Dengue-Virus induzieren Antikörper, die anderen Stämmen des Virus die Infektion erleichtern [1]. Uns sollte eine Warnung sein, dass solche „infektionsfördernden Antikörper" auch für das SARS-Coronavirus beschrieben wurden, das ein enger Verwandter des Sars-CoV-2-Virus ist. Abgesehen davon gibt es nicht genug Seren für eine passiven Impfung aller Patienten einer Pandemie. Als Alternative stehen monoklonale Antikörper zur Verfügung, die von Georges J. F. Köhler und César Milstein erstmals hergestellt wurden (Nobelpreis 1984). Damit – und mit der Technik der später entwickelten rekombinanten Antikörpern (Nobelpreis 2018 für Gregory Winter) [2] – können mittlerweile unbegrenzte Mengen von monoklonalen menschlichen Antikörpern hergestellt werden, die gegen ein Virus oder auch eine Tumorzelle gerichtet sind. Wenn diese Antikörper in Zellkultur den Eintritt des Virus verhindern, dann ist die Wahrscheinlichkeit groß, dass sie die Infektion effizient bekämpfen. Allerdings wäre zusätzlich das Wissen hilfreich, welche Antikörper häufig bei Patienten vorkommen, die nur wenige Krankheitssymptome aufweisen, bzw. welche Antikörper fehlen, wenn ein Patient schwer erkrankt – denn dadurch werden weitere mögliche Ziele für eine antikörperbasierte Therapie aufgezeigt.

### Wie findet man die „richtigen" Antikörper und Impfstoffe?

Ein kleines Virus wie das Sars-CoV-2 schränkt die Suche schon dadurch ein, dass diagnostische oder therapeutische Antikörper gegen eines von nur wenigen Virusproteinen gerichtet sein müssen. Dieser Suchraum wird erheblich größer, wenn Antikörper gegen ein bakterielles Pathogen oder auch Autoantikörper gegen menschliche Ziele gesucht werden. Noch schwieriger wird es, wenn zwar ein diagnostizierbares Krankheitsbild vorliegt (z. B. *Chronique Fatigue*-Syndrom), aber nichts darüber bekannt ist, ob diese Krankheit eine Infektion oder Autoimmunreaktion (mit)verursacht hat – und wenn ja, welche.

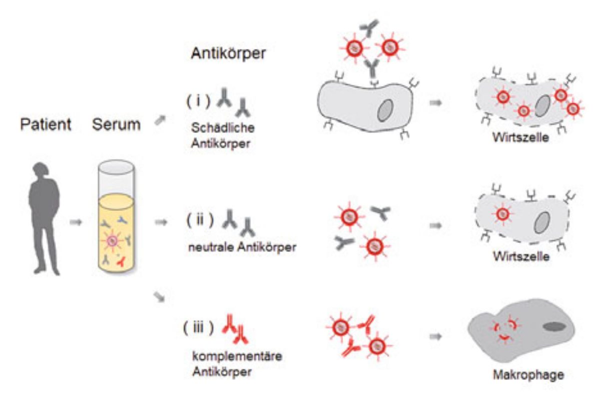

▲ **Abb. 1:** Als Reaktion auf eine Infektion bilden Patienten drei Kategorien von Antikörpern: (i) Schädliche Antikörper fördern den Eintritt der Viren in die Wirtszelle, (ii) neutrale Antikörper verhindern die Infektion der Wirtszelle nicht und (iii) nützliche Antikörper markieren die Viren, z. B. für den Abbau durch Makrophagen.

In den letzten Jahren sind immer mehr Techniken entwickelt worden, die es erlauben die „richtigen" Antikörper zu finden, nachzubauen und ihre Bindungseigenschaften zu charakterisieren. Aber nicht nur die Antikörper selbst können nachgebaut werden, dies gilt auch für die Antigene. Ein Meilenstein war die Entwicklung des *phage display* (Nobelpreis 2018 für George P. Smith) [3]. Dabei wird ein Peptid mit einem Hüllprotein des Phagen fusioniert, sodass es auf der Oberfläche des Phagen verankert wird, während gleichzeitig das dafür codierende Gen mit verpackt wird. Dadurch tragen alle so exprimierten Fusionspeptide ihr eigenes Gen „huckepack". Mittlerweile gibt es Peptidbibliotheken, die Billionen verschiedener Peptide in einem kleinen Eppendorf-Röhrchen enthalten. Der dadurch verfügbare riesige Suchraum wurde z. B. dafür verwendet, diagnostische Antikörper zu finden, die ein Prostatakarzinom anzeigen [4]. Eine Variante dieses Verfahrens führte zu den oben bereits erwähnten rekombinanten Antikörpern: An Stelle von Peptiden wurden *single chain*-Antikörper auf der Phagenhülle exprimiert [2].

### Mit einem Peptidarray können sehr viele Diagnosen gleichzeitig gestellt werden

Mitte der 80er-Jahre entwickelte Roger Ekins das Konzept des „Arrays". Dabei werden viele unterschiedliche Moleküle auf einem zweidimensionalen Träger an jeweils festgelegten Orten aufgebracht, sodass der Ort einer Bin-

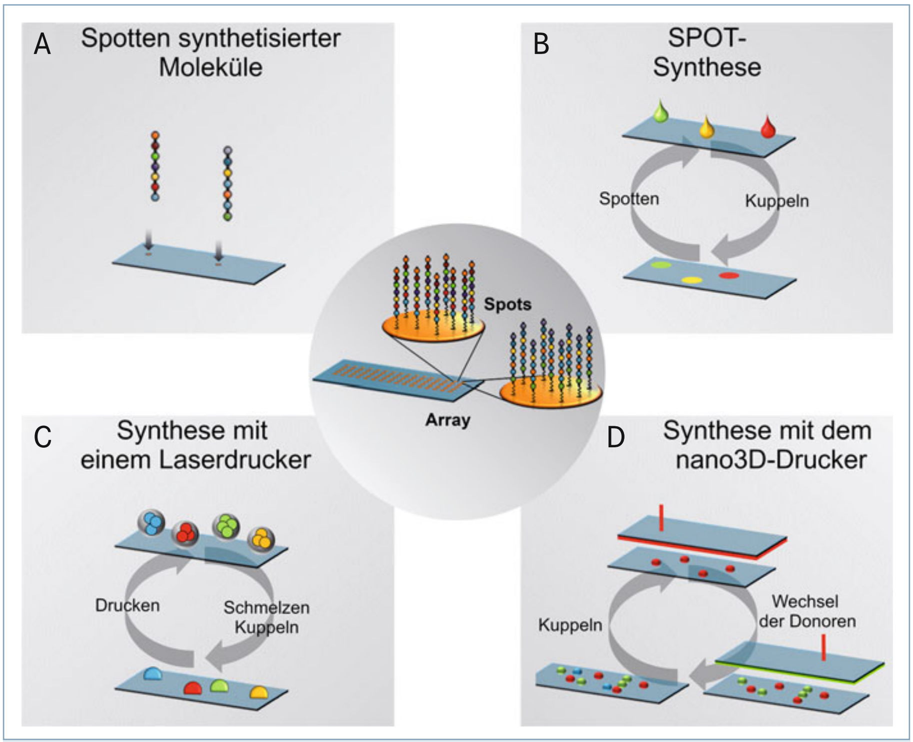

▲ **Abb. 2:** Synthesemethoden für Peptidarrays. **A**, Spotten zuvor synthetisierter Peptide auf Trägeroberflächen. **B**, SPOT-Synthese: schrittweiser Aufbau der Peptide durch Spotten von gelösten Aminosäuren. **C**, **D**, Festmaterial-basierte Synthese. Bei Raumtemperatur feste Aminosäure-Tonerpartikel werden mit einem Laserdrucker verdruckt (C). Einzelne Laserpulse eines nano3D-Druckers übertragen die in ein festes Material eingebetteten Aminosäuren auf den Array (D). Danach werden diese aufgeschmolzen, was die Kupplung der Aminosäuren ermöglicht. Bildmitte: fertiger Peptidarray.

dungsreaktion sofort aufzeigt, welches Molekül gebunden hat [5]. Mittlerweile kann man zigtausende verschiedene Proteine im Arrayformat herstellen und mit einem einzigen Experiment herausfinden, welche davon z. B. an die Antikörper eines Patienten binden [6]. Noch erfolgreicher war eine weitere Strategie, die auf der von Bruce Merrifield erfundenen Festphasensynthese von Oligonukleotiden und Peptiden beruht (Nobelpreis 1984). Dabei ist das wachsende Oligomer kovalent an einen Träger gekuppelt, sodass alle nicht umgesetzten Monomer-Bausteine einfach weggewaschen werden können. Ronald Frank parallelisierte diese Merrifield-Synthese dadurch, dass er die chemisch aktivierten Bausteine für die Peptidsynthese kombinatorisch an festgelegten Stellen auf einen Träger spottete. Die an diesen Orten synthetisierten Peptide werden dadurch jeweils um einen weiteren Baustein verlängert [7, 8]. Damit stand eine sehr einfache Methode zur Verfügung, mit der sehr viele Peptide im Arrayformat hergestellt werden konnten, ohne dass zigtausende von diagnostischen Molekülen einzeln synthetisiert, gereinigt und auf den Träger gespottet werden mussten.

Seither sind mehrere Varianten dieser Methode entwickelt worden, die sich nur darin unterscheiden, wie die chemisch aktivierten Bausteine an den Syntheseort gelangen (**Abb. 2**). Im Jahr 1993 kam der erste SPOT-Roboter auf den Markt (ABIMED Analysen, ASP222), der es erlaubte, etwa 25 Peptide pro $cm^2$ zu synthetisieren. S. Fodor *et al.* nutzten für die Computerchipherstellung entwickelte lithografische Methoden, um hochdichte Peptidarrays herzustellen. Das Licht spaltet eine Schutzgruppe ab, sodass die wachsenden Peptide nur an den durch lithografische Masken definierten Orten mit einem chemisch aktivierten Baustein verlängert werden [9]. Dieses Verfahren brachte von der Firma Affymetrix kommerzialisierte Oligonukleotidarrays hervor, aber analog hergestellte Peptidarrays hatten nie einen großen Markterfolg. Dies liegt daran, dass bei diesem Verfahren jeder Baustein nacheinander an die vorher durch die Lichtmasken definierten Orte kuppeln muss, d. h. für einen Array mit 15-mer-Peptiden mit seinen 20 verschiedenen Aminosäure-Bausteinen werden 15 × 20 = 300 Kupplungszyklen benötigt, die zu Qualitätsmängeln führen. Die Firma PEPperPRINT nutzt einen 24-Farben-Laserdrucker, um die verschiedenen in Tonerpartikel eingebetteten Aminosäure-Bausteine an die Syntheseorte zu drucken [10]. Feste Materialien „kriechen“ nicht wie Flüssigkeiten auf der Trägeroberfläche entlang, die darin eingebetteten Aminosäuren diffundieren nicht vom Syntheseort weg und sie verdunsten auch nicht, wie dies kleine flüssige Tröpfchen tun. Erst nachdem alle Aminosäure-Bausteine auf den Träger gedruckt sind, werden diese durch Hitze oder Lösungsmitteldampf verflüssigt. Dadurch können diese zu ihrem Reaktionsort auf der Trägeroberfläche diffundieren, wodurch alle Kupplungsreaktionen gleichzeitig gestartet werden. In einem anderen Ansatz wurden elektrisch geladene „Aminosäurepartikel“ von gegenpolig geladenen Pixelelektroden eines Computerchips angezogen [11]. In einer weiteren Variante dieser „Festmaterial-basierten Synthesemethode“ werden die Aminosäure-Bausteine als dünne Schicht über eine lichtabsorbierende Folie gezogen und dann an ausgewählten Bereichen mit einzelnen Laserpulsen auf einen Akzeptor übertragen [12]. Damit können derzeit etwa 17.000 Peptide pro $cm^2$ hergestellt werden, wodurch es möglich wird, ganze Bakteriengenome in die entsprechenden „Peptidome“ zu übersetzen.

## Ausblick: Antikörperseren als Informationsquelle

Menschen können zu einem gegebenen Zeitpunkt nur maximal tausend verschiedene Antikörper in therapeutisch wirksamen Mengen von etwa fünf bis zehn Mikrogramm pro Milliliter vorhalten. Die Dynamik des Auf- und Abbaus der Antikörper hat also einen evolutionären Sinn: In unserem Körper wird ständig Platz geschaffen für neue Antikörper gegen drohende Infektionen. Diese vom Immunsystem ausgewählten „sinnvollen“ Antikörper sichern der Population als Ganzes das Überleben. Wie können die oben beschriebenen Techniken genutzt werden, um herauszufinden welche Antikörper uns besonders gut schützen oder auch schaden? In Zukunft werden die Proteome wichtiger Pathogene und des Menschen im Arrayformat herstellbar sein, sodass ein einziges Experiment genügt um herauszufinden, an welche Peptide oder Proteine die Antikörper eines Menschen binden. Damit kann auch nachgefragt werden, ob es Unterschiede gibt zwischen den Menschen, die eine Krankheit gut oder schlecht überstanden haben. Diese

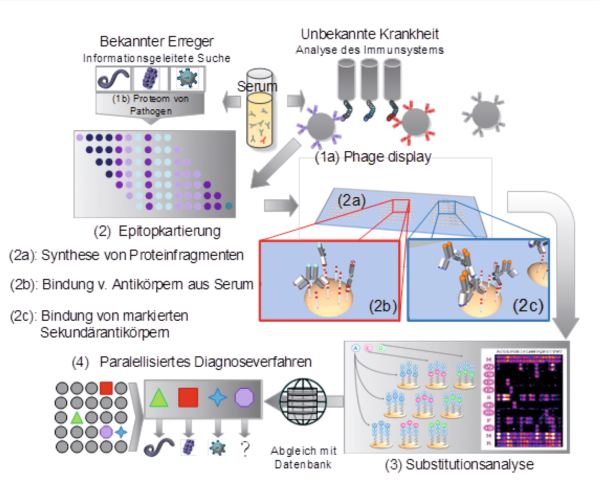

▲ **Abb. 3:** *Phage display* (1a) bietet so viele Peptide an, dass für fast jeden Antikörper eines Serums ein Binder dabei ist. Wenn die so gefundenen Peptide als Peptidarray (2a) hergestellt werden, geben nur die vervielfältigten Antikörper eines Serums (2b) ein Bindesignal (2c). Ist das Pathogen bereits bekannt, kann alternativ direkt das Peptidom des Erregers im Arrayformat hergestellt (2) und mit Patientenseren gefärbt werden (2b, 2c). Um herauszufinden, genau welche Aminosäuren innerhalb des bindenden Peptids für die Bindung wichtig sind, wird jede Position des ursprünglich gefundenen Peptids gegen alle Aminosäuren substituiert und erneut mit dem Serum gefärbt (3). In Datenbanken kann dadurch das Pathogen identifiziert werde, das dieses Antigen produziert. Mit Peptidarrays können viele Krankheiten gleichzeitig diagnostiziert werden. Das „?“ symbolisiert bisher nicht entdeckte, natürliche und regulatorische Antikörper, die so gefunden werden können (4). Teile dieser Abbildung wurden dankenswerter Weise von Wiley zur Verfügung gestellt.

Unterschiede zeigen auf, welche Antikörper Kandidaten für Therapeutika sind bzw. welche Antigene sich besonders gut für einen Impfstoff eignen (**Abb. 3**).

Anstatt zu fragen, welche Proteine eines Pathogens von Antikörpern erkannt werden, kann auch umgekehrt gefragt werden, was die Antikörper eines Menschen binden und welche dieser Antikörper nur bei erkrankten Patienten auftauchen. Laura Weber *et al.* nutzten die riesige Zahl der mittels *phage display* präsentierten Peptide, um zunächst eine Liste von Peptiden zu erhalten, die an irgendeinen Antikörper eines Patientenserums gebunden hatten. Im nächsten Schritt wurden die so gefundenen Peptide im Arrayformat synthetisiert und schließlich wurden die auch in diesem Format gebundenen Peptide systematisch an jeder Position variiert. Damit kann man herausfinden, welche Aminosäuren des Peptids für die Bindung an den Antikörper nötig sind und man kann auch fragen, welche Pathogene die Proteine codieren, die zu den jeweiligen Antikörperspezies „passen“ (**Abb. 3**). Die nachfolgenden Versuche ergaben ein überraschendes Ergebnis: Viele der so gefundenen Antikörper gibt es genau so auch bei anderen Patienten [13]. Einige dieser ubiquitären Antikörper haben offensichtlich die Aufgabe, uns gegen gefährliche Pathogene zu verteidigen, während andere regulatorische Funktionen [14] haben könnten, indem sie besonders wichtige Proteine vor einer Immunattacke schützen oder Zelltrümmer markieren, damit diese „weggeräumt“ werden können.

Es ist auch absehbar, dass die immer billiger werdenden Peptidarrays mit *point-of-care*-Systemen verbunden werden [15] und so früher oder später dazu dienen werden, sehr kostengünstig viele Diagnosen gleichzeitig zu erstellen. Sobald solche parallelisierten Diagnosen häufig geschehen, werden fast zwangsläufig Querverbindungen zwischen Krankheitsbildern und angefärbten Antigenmustern entdeckt werden. Bei etwa der Hälfte der bekannten Krankheitsbilder ist die Ursache nicht bekannt und es wird spannend sein herauszufinden, ob es bei diesen Krankheitsbildern Antikörper gibt, die auf eine Infektion oder eine Autoimmunreaktion hindeuten.


Funding Open Access funding provided by Projekt DEAL.

Danksagung

Unsere Arbeit wurde finanziert durch das BMBF (VIP+ ANTIBIOTIKA, Förderkennzeichen 03VP02840). Wir möchten uns auch bei Karin Herbster für die vielfältige Unterstützung bedanken.


Literatur

[1] Halstead SB Mahalingam S Marovich MA Intrinsic antibody-dependent enhancement of microbial infection in macrophages: disease regulation by immune complexes Lancet Infect Dis 2010 10 712 722 10.1016/S1473-3099(10)70166-3 20883967 PMC3057165
[2] McCafferty J Griffiths AD Winter G Chiswell DJ Phage antibodies: filamentous phage displaying antibody variable domains Nature 1990 348 552 554 10.1038/348552a0 2247164
[3] Smith GP Filamentous fusion phage: novel expression vectors that display cloned antigens on the virion surface Science 1985 228 1315 1317 10.1126/science.4001944 4001944
[4] Wang Y Han KJ Pang XW Large scale identification of human hepatocellular carcinoma-associated antigens by autoantibodies J Immunol 2002 169 1102 1109 10.4049/jimmunol.169.2.1102 12097419
[5] Ekins RP Multi-analyte immunoassay J Pharm Biomed Anal 1989 7 155 168 10.1016/0731-7085(89)80079-2 2488616
[6] He M Stoevesandt O Taussig MJ In situ synthesis of protein arrays Curr Opin Biotechnol 2008 19 4 9 10.1016/j.copbio.2007.11.009 18207731
[7] Southern EM Maskos U Elder JK Analyzing and comparing nucleic acid sequences by hybridization to arrays of oligonucleotides: evaluation using experimental models Genomics 1992 13 1008 1017 10.1016/0888-7543(92)90014-J 1380482
[8] Frank R Spot-synthesis: an easy technique for the positionally addressable, parallel chemical synthesis on a membrane support Tetrahedron 1992 48 9217 9232 10.1016/S0040-4020(01)85612-X
[9] Fodor S Read J Pirrung M Light-directed, spatially addressable parallel chemical synthesis Science 1991 251 767 773 10.1126/science.1990438 1990438
[10] Stadler V Felgenhauer T Beyer M Combinatorial synthesis of peptide arrays with a laser printer Angew Chem Int Ed 2008 47 7132 7135 10.1002/anie.200801616 18671222
[11] Beyer M Nesterov A Block I Combinatorial synthesis of peptide arrays onto a microchip Science 2007 318 1888 10.1126/science.1149751 18096799
[12] Loeffler FF Foertsch TC Popov R High-flexibility combinatorial peptide synthesis with laser-based transfer of monomers in solid matrix material Nat Commun 2016 7 11844 10.1038/ncomms11844 27296868 PMC4911634
[13] Weber LK Palermo A Kügler J Single amino acid fingerprinting of the human antibody repertoire with high density peptide arrays J Immunol Methods 2017 443 45 54 10.1016/j.jim.2017.01.012 28167275
[14] Rosser EC Mauri C Regulatory B Cells: Origin, Phenotype, and Function Immunity 2015 42 607 612 10.1016/j.immuni.2015.04.005 25902480
[15] Aeinehvand MM Weber L Jiménez M Elastic reversible valves on centrifugal microfluidic platforms Lab on a Chip 2019 6 1090 1100 10.1039/C8LC00849C 30785443
